# Distribution of Abdominal Obesity and Fitness Level in Overweight and Obese Korean Adults

**DOI:** 10.1155/2014/854392

**Published:** 2014-03-02

**Authors:** Sue Kim, Ji-Young Kim, Duk-Chul Lee, Hye-Sun Lee, Ji-Won Lee, Justin Y. Jeon

**Affiliations:** ^1^Department of Family Medicine, Severance Hospital, Yonsei University College of Medicine, 250 Seongsanno, Seodaemun-gu, Seoul 120-752, Republic of Korea; ^2^Department of Sport and Leisure Studies, Yonsei University, 50 Yonsei-ro, Seodaemun-gu, Seoul 120-749, Republic of Korea; ^3^Biostatistics Collaboration Units, Department of Research Affairs, Yonsei University College of Medicine, 250 Seongsanno, Seodaemun-gu, Seoul 120-752, Republic of Korea

## Abstract

*Background.* Abdominal obesity and its relative distribution are known to differ in association with metabolic characteristics and cardiorespiratory fitness. This study aimed to determine an association between fitness level and abdominal adiposity in overweight and obese adults. *Methods.* 228 overweight and obese individuals were classified as either cardiorespiratory unfit or fit based on their recovery heart rate. Visceral adipose tissue (VAT), subcutaneous adipose tissue (SAT), the visceral-to-subcutaneous adipose tissue ratio (VAT/SAT ratio), and cardiometabolic characteristics were analyzed to examine the relationship between recovery heart rate and abdominal adiposity components. *Results.* After adjustments for age and sex, significant relationships of recovery heart rate and VAT, SAT, and VAT/SAT ratio were found; however, SAT was not significantly associated after further adjustment for body mass index (BMI) (*r* = 0.045, *P* = 0.499), whereas VAT (*r* = 0.232, *P* < 0.001) and VAT/SAT ratio (*r* = 0.214, *P* = 0.001) remained associated. Through stepwise multiple regression analyses after adjustment for age, sex, BMI, lifestyle factors, mean blood pressure, fasting glucose, HOMA-IR, lipid profiles, and hsCRP, recovery heart rate was identified as an independent variable associated with VAT (*β* = 0.204, *P* < 0.001) and VAT/SAT ratio (*β* = 0.163, *P* = 0.008) but not with SAT (*β* = 0.097, *P* = 0.111). *Conclusions.* Cardiorespiratory fitness level is independently associated with VAT and the VAT/SAT ratio but not with SAT in overweight and obese adults.

## 1. Introduction

Abdominal obesity is a major risk factor for metabolic dysregulation, leading to diabetes, hypertension, and cardiovascular diseases in overweight and obese individuals, beyond overall amount of body fat [1, 2]. Accordingly, to assess abdominal adiposity, waist circumference and the waist-to-hip ratio are commonly used over body mass index (BMI) [3]. However, these anthropometric measures contain little information regarding the anatomical location of stored excess fat, which contributes to abdominal obesity and metabolic consequences. The key components of abdominal obesity are visceral adipose tissue (VAT) and subcutaneous adipose tissue (SAT), which are known to differ in their structural composition, function, and metabolic activity [4].

Visceral fat accumulation undergoes more unfavorable adverse metabolism than subcutaneous fat [5]. Previous studies have indicated that VAT demonstrates a stronger association with metabolic disturbances and cardiovascular risks than SAT [6, 7]. On the other hand, subcutaneous fat is thought to have protective properties as an adipose tissue depot [8]. Moreover, the visceral-to-subcutaneous adipose tissue ratio (VAT/SAT ratio), a measure to quantify abdominal fat distribution with regard to the propensity to store excess fat viscerally rather than subcutaneously, was found to be a correlate of cardiometabolic risk [9, 10].

Cardiorespiratory or aerobic fitness refers to the ability of the circulatory and respiratory systems to supply oxygen to muscles and organs during continuous physical activity without tiring and can be measured by recovery heart rate after exercise [11, 12]. The association of low level of cardiorespiratory fitness and high VAT, as well as high prevalence of metabolic abnormalities, was shown in several previous studies [13, 14]. Furthermore, a recent meta-analysis demonstrated that a decrease in visceral adipose tissue can be obtained by exercise in overweight adults [15]. However, few studies have examined correlations between fitness level and abdominal adipose tissue distribution in overweight and obese individuals at present.

The purpose of this study was to assess the association between cardiorespiratory fitness level expressed as recovery heart rate and VAT, SAT, and the VAT/SAT ratio measured by computed tomography (CT) scan in healthy overweight and obese adults.

## 2. Materials and Methods

### 2.1. Study Participants

Two hundred twenty-two men and women were enrolled in this subsection of the Korean Physical activity and Obesity Program (K-POP) study, which is an ongoing study designed to evaluate cardiorespiratory fitness and metabolism in overweight and obese adults in Seoul, Korea. The participants were recruited from visitors to the Obesity Clinic in the Department of Family Medicine at Severance Hospital from January 2011 to March 2013. This study complied with the Declaration of Helsinki and was approved by the Institutional Review Board of Severance Hospital.

Overweight was defined as having a BMI greater than or equal to 23 kg/m^2^, and obesity was defined as having a BMI greater than or equal to 25 kg/m^2^, following Asian-Pacific population-specific BMI criteria after consultation with a World Health Organization expert [16]. Among the overweight and obese participants, subjects aged 18 to 70 years, without history of diabetes, hypertension, dyslipidemia, or chronic liver disease, were included in the study. Also, those taking medications that affect cardiometabolic activity, such as antiobesity drugs, hypoglycemic agents, or drugs lowering blood pressure, were also excluded from the study.

Individuals who were unable to complete the cardiorespiratory fitness evaluation due to their physical or psychological conditions were excluded as well.

### 2.2. Clinical and Anthropometric Evaluation

BMI was calculated with dividing weight by square of height (kg/m^2^). Body weight was measured to the nearest 0.1 kg with an electronic scale, and height was measured to the nearest 0.1 cm with a stadiometer. Waist circumference was measured midway between the lowest rib and the iliac crest; hip circumference was measured at the widest part of the hip region in the standing position, and the waist-to-hip ratio was calculated based on these measurements. Blood pressure was measured twice by mercury sphygmomanometer after a 10 min seated rest, and mean blood pressure was calculated as [1/3 (systolic BP) + 2/3 (diastolic BP)] based on the average of the two measurements [17]. Data on past and current medical conditions and medications were collected from participants' medical records. Lifestyle factors including smoking status, alcohol consumption, and basal physical activity status were provided by participants through questionnaires. Smoking status was considered yes if the participants reported themselves to be a current smoker. Alcohol consumption was defined as a positive factor if the subjects' alcohol consumption was 72 g or more per week. Physical activity status was analyzed from a participant's overall energy expenditure calculated in metabolic equivalents- (METs-) hour per week (MET-h/week) from information collected by the Korean version of the International Physical Activity Questionnaire (IPAQ) [18].

Abdominal adipose tissue area was measured by CT scan (Tomoscan 350; Philips, Mahwah, NJ, USA). Specifically, a 10 mm CT slice scan was acquired at the L4-L5 level with subjects in the supine position to measure total abdominal tissue (TAT) and VAT areas. VAT was quantified by defining the intra-abdominal cavity at the internal aspect of the abdominal and oblique muscle walls surrounding the cavity and the posterior aspect of the vertebral body. The SAT area was calculated by subtracting the VAT area from the TAT area. The VAT/SAT ratio was calculated using these measured areas. The coefficients of variation for inter- and intraobserver reproducibility were 1.4% and 0.5%, respectively.

### 2.3. Biochemical Analyses

Biochemical analyses were performed on blood samples collected after an overnight fast (>12 hrs). Serum levels of glucose, total cholesterol, triglycerides, high-density lipoprotein cholesterol (HDL-cholesterol), low-density lipoprotein cholesterol (LDL-cholesterol), and highly sensitive C-reactive protein (hsCRP) were measured with Hitachi 7600 Automatic analyzer (High-Technologies Corporation, Hitachi, Tokyo, Japan). Fasting insulin was measured by an electrochemiluminescence immunoassay using an Elecsys 2010 instrument (Roche, Indianapolis, IN, USA), and insulin resistance was estimated using the homeostasis model assessment of insulin resistance (HOMA-IR) index [(Insulin (*μ*IU/mL) × fasting glucose (mg/dL)/18)/22.5] [19].

### 2.4. Cardiorespiratory Fitness

Cardiorespiratory fitness was measured by Tecumseh step test, a standardized 3-minute step test. Participants performed 24 steps per minute for 3 minutes based on the protocol for the Tecumseh step test, maintaining a same stepping rate on a 20.3 cm high step [14]. The participants were aided by an assistant's demonstration and a metronome cadence for proper stepping technique and constant step maintenance. Heart rates were measured by a heart rate monitor (Polar-FS3C, USA) attached to the anterior chest wall of the participant. Heart rates were recorded in a seated position 1 minute prior to exercise after a minimum 5-minute rest and 1-minute rest after the completion of the 3-minute step exercise; 1-minute recovery heart rate was measured and recorded. The expectation of this test was that participants with greater cardiorespiratory fitness would have lower 1-minute postexercise recovery heart rate than those with worse cardiorespiratory fitness [20].

### 2.5. Statistical Analyses

To compare abdominal adiposity and other metabolic variables according to cardiorespiratory fitness level, participants were grouped as unfit (low fitness level) or fit (high fitness level) individuals. Fitness level was divided into high or low according to the level of recovery heart rate either lower or higher than the median value (50th percentile) of the distribution, respectively.

Data are expressed as means ± standard deviation (SD) or percentages. Normality of the variables was tested using the Kolmogorov-Smirnov test. Data between groups were compared with an independent sample *t*-test for continuous data or the Chi-square test for categorical data.

To assess the association between fitness level expressed as recovery heart rate and the distribution of abdominal adiposity, Pearson's partial correlation analyses were performed for correlations between recovery heart rate and VAT, SAT, and the VAT/SAT ratio, after adjusting for age, sex, and BMI to assess attenuation of the associations by overall amount of body fat. The analyses were also performed for men and women separately to evaluate any differences according to sex.

Additionally, stepwise method multiple linear regression analyses were used to estimate the magnitude of the independent associations of recovery heart rate and VAT, SAT, and the VAT/SAT ratio, after adjusting for age, sex, BMI, lifestyle factors (smoking, alcohol, and physical activity status), mean blood pressure, fasting glucose, HOMA-IR, lipid profiles (LDL-cholesterol, HDL-cholesterol, and triglyceride), and hsCRP. To determine independent associations of regional adiposity distribution, SAT was included in the recovery heart rate and VAT regression model, and VAT was included in the recovery heart rate and SAT regression model, but neither VAT nor SAT was included in the recovery heart rate and VAT/SAT ratio regression model, due to the collinearity of VAT and SAT with VAT/SAT ratio. In addition, enter-method multiple linear regression analyses were performed to determine the associations with inclusion of the same variables selected in the stepwise method.

Statistical significance for all analyses was set at *P* less than 0.05. Statistical analyses were performed with SPSS software (version 20.0; SPSS Inc., Chicago, IL, USA).

## 3. Results

The clinical and biochemical characteristics of the study participants, who were divided into unfit or fit groups, are given in Table 1. Unfit individuals whose recovery heart rate after step exercise was the same or above 93 beats per minute (bpm) showed significantly higher BMI and waist circumference, but no difference was found in the waist-to-hip ratio between the two groups. In addition, mean blood pressure, fasting insulin, HOMA-IR, and triglyceride level were significantly higher in the unfit group. Finally, VAT, SAT, and the VAT/SAT ratio were all also significantly higher in unfit participants compared with fit participants.

**Figure 1:**
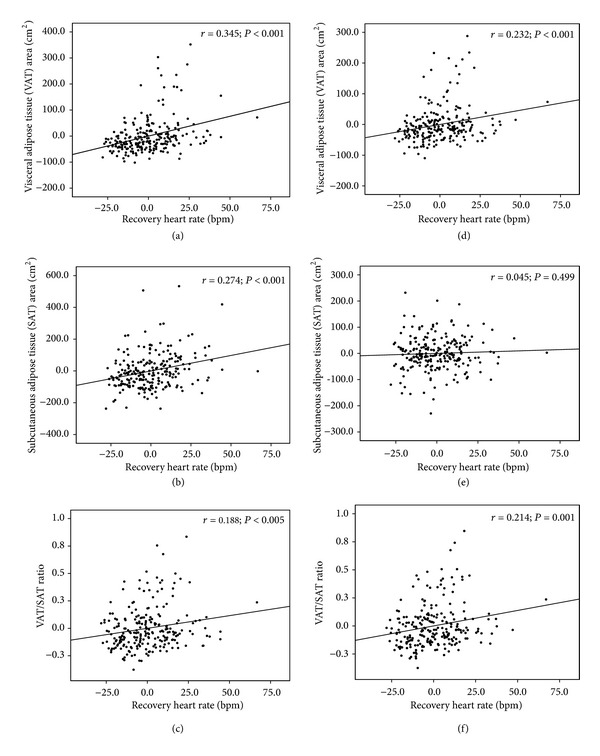
Relationship between fitness level (recovery heart rate) and abdominal adiposity (VAT, SAT, and the VAT/SAT ratio). VAT, visceral adipose tissue; SAT, subcutaneous adipose tissue; VAT/SAT ratio, visceral-to-subcutaneous adipose tissue ratio; *r*: Pearson's partial correlation coefficient (*r* = 0: no linear relationship, *r* = 1 or −1: perfect linear relationship). *x*-axes are based on calculated residuals from regressing fitness level (recovery heart rate) on age and sex (a, b, and c) and for age, sex, and BMI (d, e, and f). *y*-axes are based on calculated residuals from regressing abdominal adiposity (VAT, SAT, and the VAT/SAT ratio) on age and sex (a, b, and c) and for age, sex, and BMI (d, e, and f).

In correlation analyses, the significantly positive relationships between insulin resistance markers (fasting insulin and HOMA-IR), lipid profiles (triglyceride and HDL), and recovery heart rate were found after adjusting for age and sex (data not shown). In addition, these cardiometabolic variables were shown to be significantly correlated with abdominal adiposity (VAT, SAT, and the VAT/SAT ratio) after adjusting for age, sex, and BMI (Table 2). Although significant correlations with recovery heart rate and VAT, SAT, VAT/SAT ratio were found after adjustment for age and sex, the association between SAT and recovery heart rate was no longer significant after adjustments were made for age, for sex, and further for BMI (*r* = 0.045; *P* = 0.499), whereas significant correlations were maintained between recovery heart rate and VAT (*r* = 0.232; *P* < 0.001) and the VAT/SAT ratio (*r* = 0.214; *P* = 0.001) (Figure 1).

In addition, significant associations between recovery heart rate and VAT and VAT/SAT ratio but not SAT were also seen when analyzed separately in men: VAT (*r* = 0.340; *P* = 0.001), SAT (*r* = 0.102; *P* = 0.325), and VAT/SAT ratio (*r* = 0.288; *P* = 0.005), respectively (see Supplementary Table 1 in Supplementary Material available online at http://dx.doi.org/10.1155/2014/854392). However, in women, the results were not significant, but only with similar trend found in the relationships: VAT (*r* = 0.162; *P* = 0.066), SAT (*r* = 0.022; *P* = 0.804), and VAT/SAT ratio (*r* = 0.170; *P* = 0.050), respectively (see Supplementary Table 2).

Independent associations of recovery heart rate with regional abdominal adiposity variables were assessed in multivariable adjusted models by stepwise multiple regression analyses. Recovery heart rate was identified as a significant independent variable associated with VAT (*β* = 0.204; *P* < 0.001) and the VAT/SAT ratio (*β* = 0.163; *P* = 0.008), but not with SAT (*β* = 0.097; *P* = 0.111). Furthermore, significant associations of recovery heart rate with VAT (*β* = 0.203; *P* < 0.001) and the VAT/SAT ratio (*β* = 0.163; *P* = 0.008), but not with SAT (*β* = 0.038; *P* = 0.337), were maintained in enter-method multiple regression analyses after adjusting for the same covariates selected in the stepwise analyses (Table 3).

## 4. Discussion

In this cross-sectional study, we demonstrate that VAT and SAT, comprising abdominal adiposity, differently associate with fitness level expressed as recovery heart rate after adjusting for possible confounding factors including BMI [21]. Additionally, the VAT/SAT ratio, a measure of abdominal fat distribution between visceral and subcutaneous compartments, was also associated with recovery heart rate as was VAT in healthy overweight and obese Korean adults. Comparing the association between cardiorespiratory fitness level and abdominal obesity distribution measures, our study findings suggest that VAT is a better correlate with cardiorespiratory fitness than is SAT and that the VAT/SAT ratio is also a significant correlate with fitness.

In many previous studies, excess accumulation of VAT was indicated as a strong correlate for deteriorated metabolic health and unfavorable cardiorespiratory fitness level [22–24]. Moreover, visceral fat was shown to be a critical determinant in associations of fitness and metabolic risk modification [15]. On the other hand, even though high SAT accumulation is seen in the obese population, SAT is known to have no or a less-strong independent association with cardiometabolic factors and physical activity level than has VAT [25–27]. Furthermore, the ability to retain fat in the subcutaneous compartment, especially in the superficial layer, is suggested to be beneficial for the obese population [28]. In conditions of chronic energy balance and thus obesity, SAT has been proposed to act as an energy sink for superfluent energy, protecting ectopic fat accumulation in organs such as abdominal visceral compartments and the liver [29]. Dysfunction of SAT and excess VAT energy stores (unfavorable fat partitioning) results in an unfavorable metabolic profile [29, 30]. As a result, the VAT/SAT ratio is suggested to be a reflection of the relative distribution of abdominal fat and predict metabolic health [9, 10, 31].

Regular physical activity is known to be a favorable modifier of metabolic disease risk factors by attenuating positive energy balance and leading to reductions in weight loss and abdominal adiposity [32]. Several previous studies reported that cardiorespiratory fitness is associated with a reduced prevalence of metabolic diseases [33, 34]. In fact, by examining abdominal adipose tissue composition, fitness is more related to visceral fat reduction, even in the absence of weight loss [32, 35]. The distinctive beta-adrenergic responsiveness of VAT is a suggested mechanism to explain the selective and greater mobilization of adipose tissue from the visceral compartment than the subcutaneous region, which is driven by the sympathetic drive associated with exercise [21, 36, 37]. In addition, with reduced visceral adiposity, cardiorespiratory fitness is known to lessen the deleterious actions of unfavorable cytokines and adipokines released from visceral fat and improves responses to advantageous cytokines and adipokines, such as adiponectin and leptin [38, 39]. Moreover, the increase in fat-free lean mass and decrease in VAT through physical exercise are other factors affecting metabolic improvements [21, 39].

Only a few studies have explored the relationship between fitness and VAT compared with SAT and the VAT/SAT ratio. Larsen et al. [27] demonstrated that greater physical activity was associated with less visceral and subcutaneous fat, but after adjusting for BMI, the association only remained for VAT, which is consistent with the results of our study. Furthermore, Borel et al. [40] reported that beneficial effects from physical activity were specifically associated with VAT reduction compared to reductions in SAT. As our study focused on overweight and obese adults, it is particularly important to assess relationships between SAT and fitness and other biomarkers in overweight and obese populations, as SAT function is reported to be modified by increasing weight gain [25, 41]. Additionally, BMI and VAT adjustments are critical for examining independent associations between SAT and fitness, which can lead to different associations, as seen in our study as well as in a previous report [21, 27].

When the population was analyzed in subgroups divided by sex, the significant associations between recovery heart rate and VAT and VAT/SAT ratio, but not SAT, were only found in men, whereas only similar trend of the relationship was seen in women. These findings may indicate that the associations differ sex specifically, but with similar trend demonstrated in women and with cross-sectional analyses with rather small sample in our study. Thus, it is difficult to make concrete conclusions. Further prospective studies with larger samples are necessary to define the sex-specific characteristics.

There are some limitations to this study. First of all, the 3-minute step test was used to estimate cardiorespiratory fitness level and this could be a limitation since the level of adiposity would influence the heart rate; more obese participants would have increased their heart rate more due to having to repetitively lift more weight up and down on a standard-height bench. Regardless of this limitation, the 3-minute step test has been validated previously and it has been used in clinical setting and several prior epidemiologic studies [42–45]. Another limitation of the study is that the directionality and causality of the results cannot be determined with certainty in this cross-sectional design. Finally, as participants were recruited from the obesity clinic with a greater interest in health than the general population, it may be difficult to generalize the results to the public.

## 5. Conclusions

In conclusion, cardiorespiratory fitness was independently associated with VAT and the VAT/SAT ratio, but not with SAT, in overweight and obese Korean adults. Our findings collectively suggest that fitness may be a modifier of abdominal adiposity distribution, leading to favorable metabolic health. Further studies are required to clarify the clinical associations of these findings and to explore their pathophysiological significance.

## Supplementary Material

In men, significant associations between recovery heart rate and VAT and VAT/SAT ratio but not SAT were also seen when analyzed separately in men: VAT (*r*= 0.340; *P*= 0.001), SAT (*r*= 0.102; *P*= 0.325), and VAT/SAT ratio (*r*= 0.288;*P*= 0.005), respectively (Table 1).
In women, the results were not significant, but only with similar trend found in the relationships: VAT (*r*= 0.162;*P*= 0.066), SAT (*r*= 0.022;*P*= 0.804), and VAT/SAT ratio (*r*= 0.170;*P*= 0.050), respectively (Table 2).

## Figures and Tables

**Table 1:** Clinical characteristics of the study participants according to fitness level (recovery heart rate).

	Unfit (RHR ≥ 93) (*n* = 114)	Fit (RHR < 93) (*n* = 114)	*P* value^a^
Age (years)	32.46 ± 9.40	34.43 ± 9.97	0.118
Male, *n* (%)	54 (44.6)	46 (40.4)	0.507
BMI (kg/m^2^)	30.09 ± 4.70	28.25 ± 3.63	0.001
Waist (cm)	99.08 ± 12.54	94.61 ± 9.17	0.001
Waist-to-hip ratio	0.91 ± 0.055	0.90 ± 0.052	0.106
Mean BP (mmHg)	98.04 ± 12.92	92.58 ± 11.93	0.001
Alcohol, *n* (%)	35 (29.2)	34 (29.9)	0.787
Smoking, *n* (%)	23 (25.2)	26 (23.3)	0.638
Physical activity (MET-h/week)	29.61 ± 24.46	29.82 ± 24.55	0.949
Fasting glucose (mg/dL)	100.82 ± 85.89	88.76 ± 9.58	0.130
Fasting insulin (*μ*U/mL)	15.95 ± 18.75	9.78 ± 7.51	0.001
HOMA-IR	3.68 ± 4.68	2.18 ± 1.76	0.001
Cholesterol (mg/dL)	198.95 ± 37.97	195.60 ± 35.23	0.483
Triglyceride (mg/dL)	142.39 ± 135.98	105.72 ± 50.78	0.006
LDL (mg/dL)	122.68 ± 35.29	121.96 ± 34.04	0.979
HDL (mg/dL)	49.53 ± 11.37	51.50 ± 11.31	0.135
hsCRP (mg/L)	2.15 ± 2.42	1.73 ± 5.27	0.442
VAT area (cm^2^)	142.35 ± 89.47	110.75 ± 62.29	<0.001
SAT area (cm^2^)	317.51 ± 118.44	276.20 ± 99.17	0.004
VAT/SAT ratio	0.47 ± 0.25	0.39 ± 0.18	0.011
Recovery heart rate	105.96 ± 12.02	81.26 ± 7.18	0.001

RHR: recovery heart rate; BMI: body mass index; BP: blood pressure; MET-h/week: metabolic equivalents-hour per week; HOMA-IR: homeostasis model assessment of insulin resistance; LDL: low-density lipoprotein; HDL: high-density lipoprotein; hsCRP: highly sensitive C-reactive protein; VAT: visceral adipose tissue; SAT: subcutaneous adipose tissue; VAT/SAT ratio: visceral-to-subcutaneous adipose tissue ratio.

Values are expressed as means ± SD for continuous variables or % for categorical variables.

^a^ *P* values are calculated by an independent sample *t-*test for continuous variables or the Chi-square test for categorical variables.

**Table 2:** Correlation coefficients between fitness level (recovery heart rate) and cardiometabolic characteristics and abdominal adiposity (VAT, SAT, and the VAT/SAT ratio).

	VAT	SAT	VAT/SAT ratio
Waist (cm)	0.273**	0.640**	0.008
Waist-to-hip ratio	0.230**	0.159*	0.110
Mean BP (mmHg)	−0.054	−0.006	0.005
Physical activity (MET-h/week)	−0.027	0.013	0.015
Fasting glucose (mg/dL)	0.009	0.005	−0.031
Fasting insulin (*μ*U/mL)	0.115**	0.018	0.128
HOMA-IR	0.102**	0.003	0.109
Cholesterol (mg/dL)	0.083	0.111	0.019
Triglyceride (mg/dL)	0.104*	0.034	0.145*
LDL (mg/dL)	0.054	0.067	0.017
HDL (mg/dL)	−0.040	−0.202	−0.197**
hsCRP (mg/L)	−0.074	0.007	−0.076
Recovery heart rate	0.232**	0.045	0.214**

VAT: visceral adipose tissue; SAT: subcutaneous adipose tissue; VAT/SAT ratio: visceral-to-subcutaneous adipose tissue ratio; BMI: body mass index; BP: blood pressure; MET-h/week: metabolic equivalents-hour per week; HOMA-IR: homeostasis model assessment of insulin resistance; LDL: low-density lipoprotein; HDL: high-density lipoprotein; hsCRP: highly sensitive C-reactive protein.

*P < 0.05, **P < 0.01, calculated by Pearson's partial correlation adjusted for age, sex, and BMI.

**Table 3:** Stepwise method and enter-method multiple linear regression analyses of fitness (recovery heart rate) and other cardiometabolic characteristics and abdominal adiposity (VAT, SAT, and the VAT/SAT ratio).

	VAT		SAT		VAT/SAT ratio		VAT		SAT		VAT/SAT ratio	
	*B* (SE)	*P* value^a^	*B* (SE)	*P* value^b^	*B* (SE)	*P* value^c^	*B* (SE)	*P* value^d^	*B* (SE)	*P* value^d^	*B* (SE)	*P* value^d^
RHR	0.204 (0.264)	<0.001	0.097 (1.601)	0.111	0.163 (0.001)	0.008	0.203 (0.262)	<0.001	0.038 (0.282)	0.337	0.163 (0.001)	0.008
Age	0.396 (0.414)	<0.001			0.484 (0.001)	<0.001	0.405 (0.407)	<0.001	−0.056 (0.437)	0.142	0.488 (0.001)	<0.001
Sex			0.179 (9.243)	<0.001			−0.023 (8.444)	0.691	0.190 (9.078)	<0.001	−0.154 (0.027)	0.011
BMI (kg/m^2^)	0.429 (0.974)	<0.001	0.883 (1.093)	<0.001			0.429 (0.974)	<0.001	0.870 (1.113)	<0.001	−0.146 (0.003)	0.024
HDL (mg/dL)			0.125 (0.423)	0.004	−0.177 (0.001)	0.003	−0.030 (0.511)	0.610	0.121 (0.417)	0.004	−0.177 (0.001)	0.003
hsCRP (mg/L)					−0.136 (0.003)	0.024	−0.078 (0.960)	0.151	0.013 (1.032)	0.735	−0.136 (0.003)	0.024

VAT: visceral adipose tissue; SAT: subcutaneous adipose tissue; VAT/SAT ratio: visceral-to-subcutaneous adipose tissue ratio; RHR: recovery heart rate; BMI: body mass index; HDL: high-density lipoprotein; hsCRP: highly sensitive C-reactive protein.

^a,b,c^ *P* values are calculated by stepwise method multiple linear regression analyses.

^a^Model variables included age, sex, BMI, smoking, alcohol, physical activity status, mean BP, fasting glucose, HOMA-IR, lipid profiles (LDL, HDL, and triglyceride), hsCRP, and SAT.

^b^Model variables included age, sex, BMI, smoking, alcohol, physical activity status, mean BP, fasting glucose, HOMA-IR, lipid profiles (LDL, HDL, and triglyceride), hsCRP, and VAT.

^c^Model variables included age, sex, BMI, smoking, alcohol, physical activity status, mean BP, fasting glucose, HOMA-IR, lipid profiles (LDL, HDL, and triglyceride), and hsCRP.

^d^ *P* values are calculated by enter-method multiple linear regression analyses; variables included age, sex, BMI, HDL, and hsCRP.
