# Supplementary material for: Distribution of Abdominal Obesity and Fitness Level in Overweight and Obese Korean Adults
Source: Int J Endocrinol. 2014 Mar 2;2014:854392. doi: 10.1155/2014/854392 (PMC3958687; doi:10.1155/2014/854392)
Supplement: Supplementary file 1 — In men, significant associations between recovery heart rate and VAT and VAT/SAT ratio but not SAT were also seen when analyzed separately in men: VAT (r= 0.340; P= 0.001), SAT (r= 0.102; P= 0.325), and VAT/SAT ratio (r= 0.288;P= 0.005), respectively (Table 1). In women, the results were not significant, but only with similar trend found in the relationships: VAT (r= 0.162;P= 0.066), SAT (r= 0.022;P= 0.804), and VAT/SAT ratio (r= 0.170;P= 0.050), respectively (Table 2). [file 854392.f1.pdf]

Supplementary table 1.

Correlation coefficients between fitness level (recovery heart rate) and cardiometabolic characteristics and abdominal adiposity (VAT, SAT, and the VAT/SAT ratio) in men

	VAT	SAT	VAT/SAT ratio
Waist (cm)	0.373**	0.747**	0.024
Waist-to-hip ratio	0.195	0.293**	0.106
Mean BP(mmHg)	-0.014	-0.084	0.007
Physical activity (MET-h/week)	-0.036	0.034	-0.002
Fasting glucose (mg/dL)	0.200	0.218*	0.008
Fasting insulin(μU/mL)	0.103*	0.045	0.146
HOMA-IR	0.088	0.007	0.157
Cholesterol (mg/dL)	0.156	0.135	0.074
Triglyceride (mg/dL)	0.146	0.004	0.197*
LDL (mg/dL)	0.088	0.136	0.013
HDL (mg/dL)	-0.108	0.019	-0.013
hsCRP (mg/L)	0.036**	0.195	0.252*
Recovery heart rate	0.340**	0.102	0.288**

Abbreviations: VAT, visceral adipose tissue; SAT, subcutaneous adipose tissue; VAT/SAT ratio, visceral-to-subcutaneous adipose tissue ratio; BMI, body mass index; BP, blood pressure; MET-h/week, metabolic equivalents hour per week; HOMA-IR, homeostasis model assessment of insulin resistance; LDL, low-density lipoprotein; HDL, high-density lipoprotein; hsCRP, highly sensitive C-reactive protein

*p<0.05, **p<0.01, calculated by Pearson's partial correlation adjusted for age and BMI

Supplementary table 2.

Correlation coefficients between fitness level (recovery heart rate) and cardiometabolic characteristics and abdominal adiposity (VAT, SAT, and the VAT/SAT ratio) in women

	VAT	SAT	VAT/SAT ratio
Waist (cm)	0.225**	0.557**	0.040
Waist-to-hip ratio	0.279**	0.053	0.185*
Mean BP(mmHg)	0.011	-0.062	0.074
Physical activity (MET-h/week)	-0.010	0.040	0.021
Fasting glucose (mg/dL)	0.004	0.044	-0.037
Fasting insulin(μU/mL)	0.187*	0.062	0.232**
HOMA-IR	0.142	0.069	0.139
Cholesterol (mg/dL)	0.029	0.143	0.020
Triglyceride (mg/dL)	0.087	0.017	0.193*
LDL (mg/dL)	0.026	0.051	0.013
HDL (mg/dL)	-0.003	-0.318**	-0.225*
hsCRP (mg/L)	-0.015	0.165	-0.118
Recovery heart rate	0.162	0.022	0.170

Abbreviations: VAT, visceral adipose tissue; SAT, subcutaneous adipose tissue; VAT/SAT ratio, visceral-to-subcutaneous adipose tissue ratio; BMI, body mass index; BP, blood pressure; MET-h/week, metabolic equivalents hour per week; HOMA-IR, homeostasis model assessment of insulin resistance; LDL, low-density lipoprotein; HDL, high-density lipoprotein; hsCRP, highly sensitive C-reactive protein

*p<0.05, **p<0.01, calculated by Pearson's partial correlation adjusted for age and BMI
